# Assessment of Nano-Indentation Method in Mechanical Characterization of Heterogeneous Nanocomposite Materials Using Experimental and Computational Approaches

**DOI:** 10.1038/s41598-019-51904-4

**Published:** 2019-10-31

**Authors:** A. Karimzadeh, S. S. R. Koloor, M. R. Ayatollahi, A. R. Bushroa, M. Y. Yahya

**Affiliations:** 1 Centre for Advanced Composite Materials, School of Mechanical Engineering, Faculty of Engineering, Universiti Teknologi Malaysia, 81310 Johor Bahru, Johor Malaysia; 2 Institute for Nanomaterials, Advanced Technologies and Innovation, Technical University of Liberec, Studentska 2, 461 17 Liberec, Czech Republic; 3 Fatigue and Fracture Research Laboratory, Center of Excellence in Experimental Solid Mechanics and Dynamics, School of Mechanical Engineering, Iran University of Science and Technology, Narmak, Tehran, 16846 Iran; 4 Department of Mechanical Engineering, Faculty of Engineering, University of Malaya, Kuala Lumpur, 50603 Malaysia; 5 Centre of Advanced Manufacturing and Mechanical Engineering, Faculty of Engineering, University of Malaya, Kuala Lumpur, 50603 Malaysia

**Keywords:** Mechanical engineering, Characterization and analytical techniques

## Abstract

This study investigates the capacity of the nano-indentation method in the mechanical characterization of a heterogeneous dental restorative nanocomposite using experimental and computational approaches. In this respect, Filtek Z350 XT was selected as a nano-particle reinforced polymer nanocomposite with a specific range of the particle size (50 nm to 4 µm), within the range of indenter contact area of the nano-indentation experiment. A Sufficient number of nano-indentation tests were performed in various locations of the nanocomposite to extract the hardness and elastic modulus properties. A hybrid computational-experimental approach was developed to examine the extracted properties by linking the internal behaviour and the global response of the nanocomposite. In the computational part, several representative models of the nanocomposite were created in a finite element environment to simulate the mechanism of elastic-plastic deformation of the nanocomposite under Berkovich indenter. Dispersed values of hardness and elastic modulus were obtained through the experiment with 26.8 and 48.5 percent average errors, respectively, in comparison to the nanocomposite properties, respectively. A disordered shape was predicted for plastic deformation of the equilateral indentation mark, representing the interaction of the particles and matrix, which caused the experiment results reflect the local behaviour of the nanocomposite instead of the real material properties.

## Introduction

In the past few decades, the nano-indentation experiment has been considered as one of the important non-destructive test methods for the determination of the mechanical properties of bulk materials, coatings, thin films, etc.^1–12^. In the nano-indentation experiment, many tests could be conducted on a small region of a sample rather than testing of many large scale specimens. Thus, the nano-indentation experiment is viewed as an appropriate alternative for macro-scale testing in mechanical characterization of expensive advanced materials (e.g. biomaterials)^11–15^. Although, the nano-indentation experiment is a recognised method to characterize the properties of homogenous materials^3,4,11^, the method has been utilized for mechanical characterization of many heterogeneous materials^15–18^. In this respect, the heterogeneous biomaterials such as polymers reinforced with hydroxyapatite particles, epoxy/silica nanocomposites, Ti and Ti-TiB composites, dental restorative composites, etc.^19–29^ have been participated in the mechanical characterization program, while the effect of the material non-homogeneity on the nano-indentation experiment results has not been addressed in these studies. Despite the limited researches about the nano-indentation experiment on heterogeneous materials, this method has been considered valid if different parts of the non-homogeneity were in contact with the indenter during the indentation process^30–32^. While, the limit of the particle size range of the heterogeneous materials for an accurate measurement of the properties during the nano-indentation experiment has not been defined. In this respect, a nano-particle reinforced polymer nanocomposite with a specific range of particle size (50 nm to 3 µm), which is widely used in dentistry applications, is selected to assess the nano-indentation method for characterizing the behaviour of the heterogeneous material.

In clinical applications, the assessment of the mechanical behaviour of dental restorative nanocomposite materials under loading in oral conditions is  dependent to the accurate determination of the mechanical properties of the materials^33–35^. Conventional methods of material characterization are very challenging and costly due to the difficulties in the preparation of the macro size nanocomposite specimen. Therefore, the nano-indentation experiment is a good alternative for the characterization of dental restorative nanocomposites^7,13,33^. Dental nanocomposites are a kind of heterogeneous materials in the scale of the nano-indentation test. Thus, the accuracy and the limitation of the nano-indentation experiment in the mechanical characterization of dental nanocomposites, requires further investigation on the mechanism of elastic-plastic deformation under indentation process. In this respect, computational method such as finite element (FE) is recognized as an effective method to study the internal behaviour of the nanocomposite through such loading^6,36^.

The FE simulation has been employed in many researches to study on the mechanical behaviour of advanced materials under nano-indentation process^4–6,37,38^. In these studies, simulation is used to understand the mechanism of deformation of the specimen under the indentation load and provide a realization of the local and global behaviours of the material^4–6,37,38^. Some researchers evaluated the stress and strain fields around the indentation region to assess the effects of physical parameters such as the indenter and the specimen configurations on the mechanical properties of materials^5,38^. The development of such models is inexpensive and provides valuable information that is hardly acquired during the experiment^39–42^. However, the nano-indentation experiment on heterogeneous materials has been modelled in just a few studies^43–45^. Olivas *et al*.^43^ and also Pereyra and Shen^44^ employed a 2-dimensional FE model to investigate the stress distribution in Al/SiC composite through the nano-indentation. In their study, a quarter of SiC particle embedded in Al matrix was modeled without considering the effect of adjacent particles. The effect of particle dispersion beneath a spherical indenter on the indentation response of Al/SiC composites was investigated in Ekik *et al*.^45^ study, in which SiC particles with uniform sizes were distributed randomly in the matrix through an axisymmetric model. The influence of the distribution of fillers with different sizes around a non-symmetric indenter such as Berkovich on the results of the nano-indentation has not been discussed in these researches^43–45^.

The present study aims to investigate on the ability of the nano-indentation method in the mechanical characterization of heterogeneous materials. A dental restorative nanocomposite is selected as a type of heterogeneous biomaterials with a specific construction of a biopolymer reinforced with ceramic nano-particles. A sufficient number of nano-indentation experiments is performed in different locations of the dental nanocomposite to extract the mechanical response and properties of the material. A hybrid computational-experimental approach is developed to provide a detailed analysis for mechanical characterization of the material. This approach employs the experiment data in the form of global response of the nanocomposite to develop a valid 3-dimensional FE model and simulation process. Then, the validated model is used to predict the mechanism of elastic-plastic deformation of the nanocomposite under equilateral Berkovich indenter loading. Therefore, many representative models containing randomly distributed nano-particles in the matrix phase are created for the nanocomposite using FE method to predict and describe the local behaviour and the global response of the material under indentation load. The combination of the experiment and simulation data is used to provide an analysis of the mechanical behaviour of the nanocomposite. The scientific approach is suggested for investigation on the validity of the nano-indentation experiment in the characterization of heterogeneous nanocomposites with different particle sizes.

## Material and Experiment

### Sample preparation

**Figure 1:**
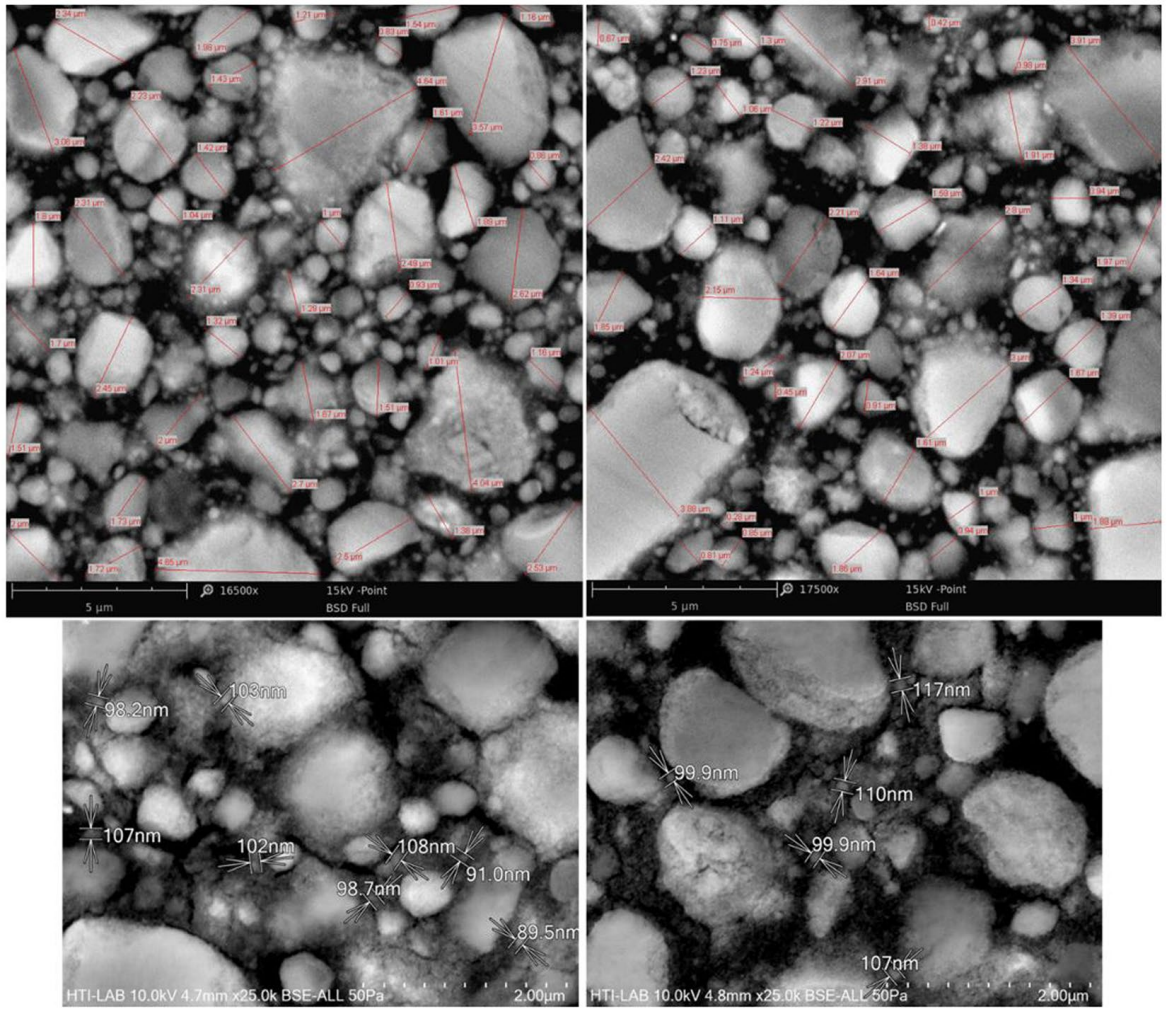
The SEM image of the Filtek Z350 XT nanocomposite with the various particle sizes.

Filtek Z350 XT (3 M ESPE, USA) nanocomposite was selected as a representative of particle reinforced dental restorative material with microstructure shown in Fig. 1. This nanocomposite composes of polymer resin and spherical ceramic particles with volume fraction of 63.3%. The resin is mainly Bis-GMA biopolymer and particles are made of the Zirconia ceramic. The particles in the size range of 50 nm to 3 µm were dispersed evenly in the resin content. In order to make specimens for the experiments, some disc-shaped samples with a diameter of 10 mm and thickness of 3 mm were prepared using Filtek Z350 XT nanocomposite; then each side of the specimens was light cured for 20 seconds using an LED light cure gun (Sony, Japan) with intensity of 400 mW/cm^2^ close to the specimen surface according to the manufacturer’s instruction of the nanocomposite. The surface of all samples was grounded using 400 to 3000 grit sand-paper and smoothed by 1, 0.5 and 0.25 microns diamond pastes, respectively. Such a process was necessary to make the sample surface very smooth for the nano-indentation experiment and scanning electron microscopy (SEM). All specimens were kept in ambient conditions of 24 °C and 40% humidity before the tests.

### Scanning electron microscopy

SEM was performed on the surface of the nanocomposite specimen to investigate about the size and distribution of the particles constituent, as shown in Fig. 1. For this purpose, the specimen was mounted on a carbon double-side tape and placed in a Phenom Prox SEM instrument (Netherlands) under 15 kv accelerated voltages. The particles with a diameter less than 100 nm were observed using Schottky field emission scanning electron microscope SU5000 (Japan) under accelerated voltage of 10 kv, in order to provide high quality nano-scale images. Then, the diameter of particles was measured using ImageJ program to determine the values of the particle diameter range around the testing region.

### Nano-indentation experiment

The nano-indentation experiment was conducted on the dental restorative nanocomposite specimens using Hysitron TI 750 Ubi L (Hysitron Inc., USA) and a Berkovich indenter tip. The test instrument was calibrated according to the approach proposed by Oliver and Pharr^46^. The nano-indentation experiment was performed in a displacement control mode in which the indenter moves with a specified rate into the sample up to a pre-set maximum indentation depth. The maximum indentation depth was set to 300 nm with the displacement rate of 10 nm/sec. Previous researches^47,48^ showed that the nano-indentation depth has significant effects on the test results, and in the polymeric materials the test results are almost stable after the indentation depth of 200 nm. On the other hand, the indentation depth should be deep enough to minimize the surface effect of the specimen, and meanwhile, it should be less enough to cause an indentation impression without any damage around it on the specimen. Since, the nanocomposite used in this study consists of a polymeric resin and ceramic particles (which are brittle materials), the indentation depth of 300 nm was selected to fulfil all of these factors about the indentation depth. Moreover, the projected area of the Berkovich indenter at 300 nm penetration is an equilateral triangle with edge size of 2.3 μm, in which a wide range of the particles of the considered nanocomposite could be loaded. At the maximum indentation depth (i.e. 300 nm), the indenter was kept for 10 sec and then the indenter is pulled out with the rate of 10 nm/sec to let the material relax. According to the previous studies^49,50^, the indenter should be held at the maximum indentation depth in order to eliminate the time dependent behaviour of the material. The duration of holding the indenter at the maximum depth was selected so that no variation was observed in the value of indentation load by increasing the holding time. Therefore, the rate of loading has no effect on the results of the experiment.

The reaction force of the specimen applied to the indenter was measured continuously throughout the test and the response of the system was recorded in the form of the load-displacement curve. Then, the system response is used to investigate the mechanical properties of the material, including the hardness and the elastic modulus. At least 20 indentations were performed on randomly selected sites of the specimen. Before and after each indentation, scanning probe microscopy (SPM) images were taken from the indentation sites to make sure that no damage occurs around the indentation impression.

#### Calculation of the mechanical properties

The hardness value in each test is calculated by dividing the maximum penetration load to the projected contact surface between the indenter and the sample as given in Eq. (1)^46^:

1 $${\rm{H}}=\,\frac{{{\rm{P}}}_{{\rm{\max }}}}{{\rm{A}}}$$

where *H* is the material hardness or indentation hardness, *P*_*max*_ is the maximum normal indentation load and *A* is the projected contact area between the indenter and the specimen., The effective elastic modulus (*E*_*eff*_) is calculated using Eq. (2) and load-displacement curve that was recorded during the nano-indentation experiment; then the elastic modulus of the material (*E*) is obtained by substituting *E*_*eff*_ in Eq. (3)^46^:

2 $${{\rm{E}}}_{{\rm{eff}}}=\frac{\sqrt{{\rm{\pi }}}}{{\rm{2}}}\frac{{\rm{dP}}}{{\rm{dh}}}\frac{{\rm{1}}}{\sqrt{{\rm{A}}}}$$

3 $$\frac{{\rm{1}}}{{{\rm{E}}}_{{\rm{eff}}}}\,=\,\frac{{1-{\rm{\nu }}}^{{\rm{2}}}}{{\rm{E}}}+\frac{{{1-{\rm{\nu }}}_{{\rm{i}}}}^{{\rm{2}}}}{{{\rm{E}}}_{{\rm{i}}}}$$

where *h* represents the indentation depth, *dP/dh* is the slope of the unloading part of the load-displacement curve at the maximum indentation depth, *E*_*i*_ and *ν*_*i*_ are the elastic modulus and Poisson’s ratio of the indenter tip, and *ν* is the Poisson’s ratio of the specimen. Considering the values of *ν*, *E*_*i*_ and *ν*_*i*_ equal to 0.31^51^, 1140 GPa^52^ and 0.07^52^, respectively, the elastic modulus of the samples is calculated from Eq. (3). The values of *E*_*i*_ and *ν*_*i*_ are obtained from the technical information of the Hysitron nano-indentation instrument^52^.

### Hybrid computational-experimental approach

**Figure 2:**
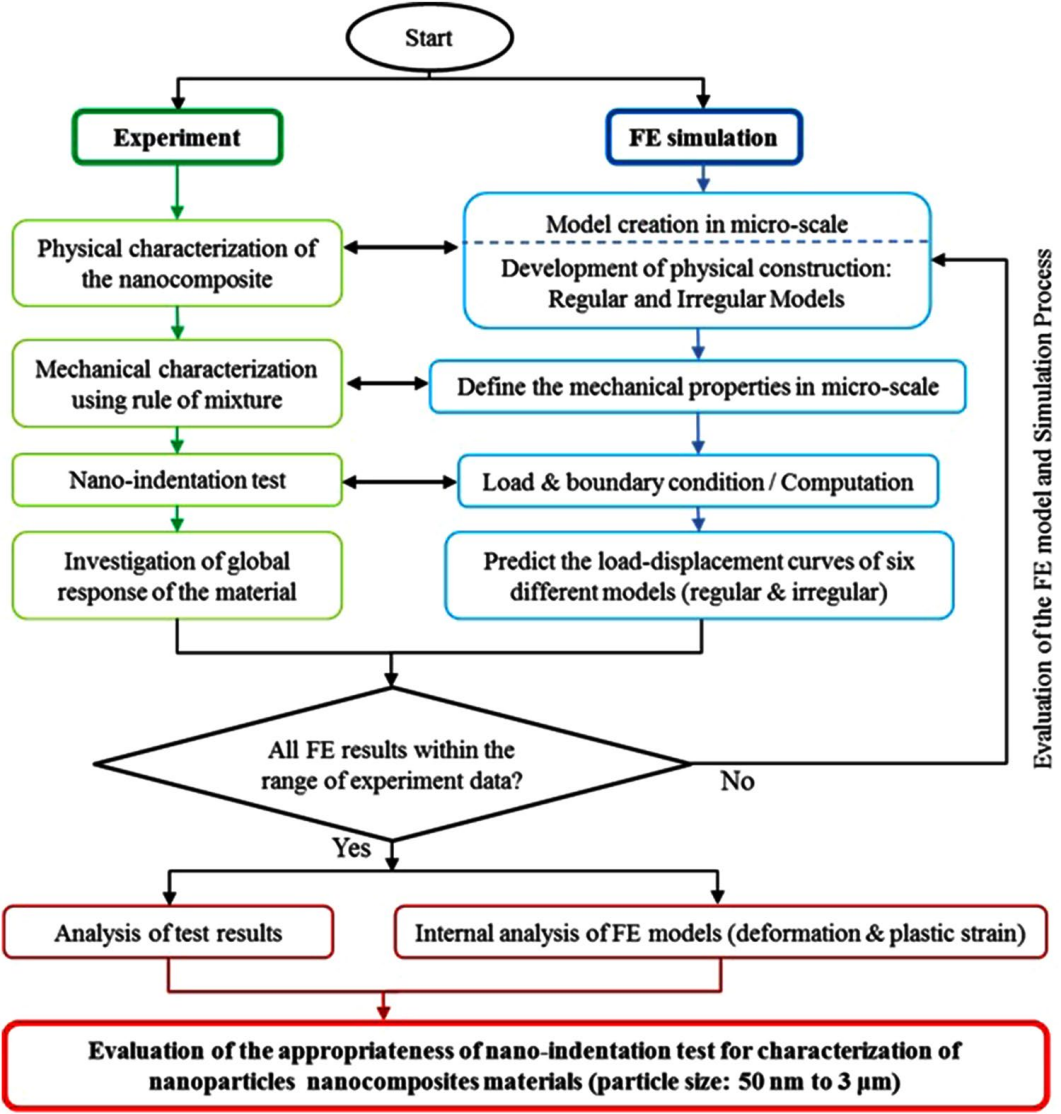
Flowchart of the hybrid computational-experimental approach.

The results of the nano-indentation experiment were used to obtain the mechanical properties of the nanocomposite. Although many numbers of tests were performed, dispersed data are obtained for the properties with negligible repeatability in the global response of the material (e.g. load-displacement curve). In this condition, assessment of the elastic-plastic behaviour and understanding the mechanism of deformation in the nanocomposite under the Berkovich indenter load would be an important task that could highlight the reasons for such responses. In this respect, FE simulation of the experiment is a recognized method to analyse the mechanical behaviour of the material^39–42^. Consequently, a computational-experimental approach is required to link the FE simulation and experiment data in order to develop a valid FE model, in which the data are used to describe the disperse response of the material that led to calculation of different mechanical properties. The flowchart of the approach is illustrated in Fig. 2.

According to Fig. 2, in the experiment part of the approach, the physical configuration of the model was identified that include recognition of each constituent, the particle size range and their volume fractions. In the mechanical characterization part, the rule of mixture as well as the literature data was used to identify the elastic-plastic properties of the nanocomposite and its constituent. The physical and mechanical information were used to build the FE model of the case with different configurations that is introduced as regular and irregular constructions. Then, the nano-indentation test was performed to obtain the global response (i.e. load-displacement curve) of the material that was also used to obtain the mechanical properties of the nanocomposite using nano-indentation method. The loading and boundary conditions were applied to control the FE models in which the predicted results were compared with experiment data. In this approach, six different FE models were built in which all global responses assume to be within the experiment results range in order to consider validation of the FE models and simulation process. The results of validated FE models including the deformation and plastic strain of the nanocomposite under Berkovich indenter were obtained through internal analysis, and used to evaluate the suitability of the nano-indentation method in the mechanical characterization of the nano-particle nanocomposite. The approach is applicable for assessment of nano-indentation method in the characterization of nanocomposites with different particle sizes.

### FE simulation of the nano-indentation experiment

**Figure 3:**
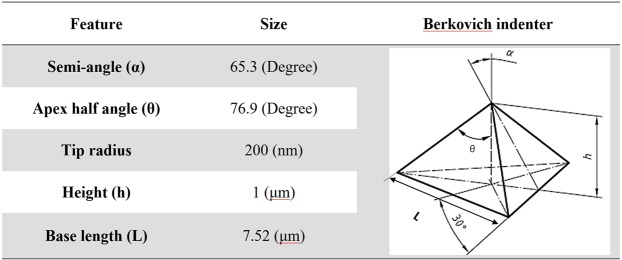
The geometrical configuration of the Berkovich indenter tip.

Several 3-dimensional FE models were developed to simulate the nano-indentation experiment on the dental nanocomposite using ABAQUS software. The Berkovich indenter tip was modelled according to the general information of a standard Berkovich tip^1,12^, as presented in Fig. 3.

A cubic block with the dimension of 4 μm was created as the representative volume element of the dental nanocomposite specimen subjected to indentation load. The block dimension was considered big enough to encompass the largest particle and also have zero deformations on the exterior surfaces of the specimen. According to the SEM images of the material, the diameter of particles is in the range of 50 nm to 4 μm as illustrated in Fig. 1. However, modelling the particles with diameters less than 0.5 μm may extremely increase the computational time. Subsequently, the effect of fine particles (d < 0.5 μm) in the nanocomposite has been seen in the FE modelling process by updating the mechanical properties of the resin based on the rule of mixture [35, 36]. Therefore, the resin region that contains fine particles is called “Matrix”.

**Figure 4:**
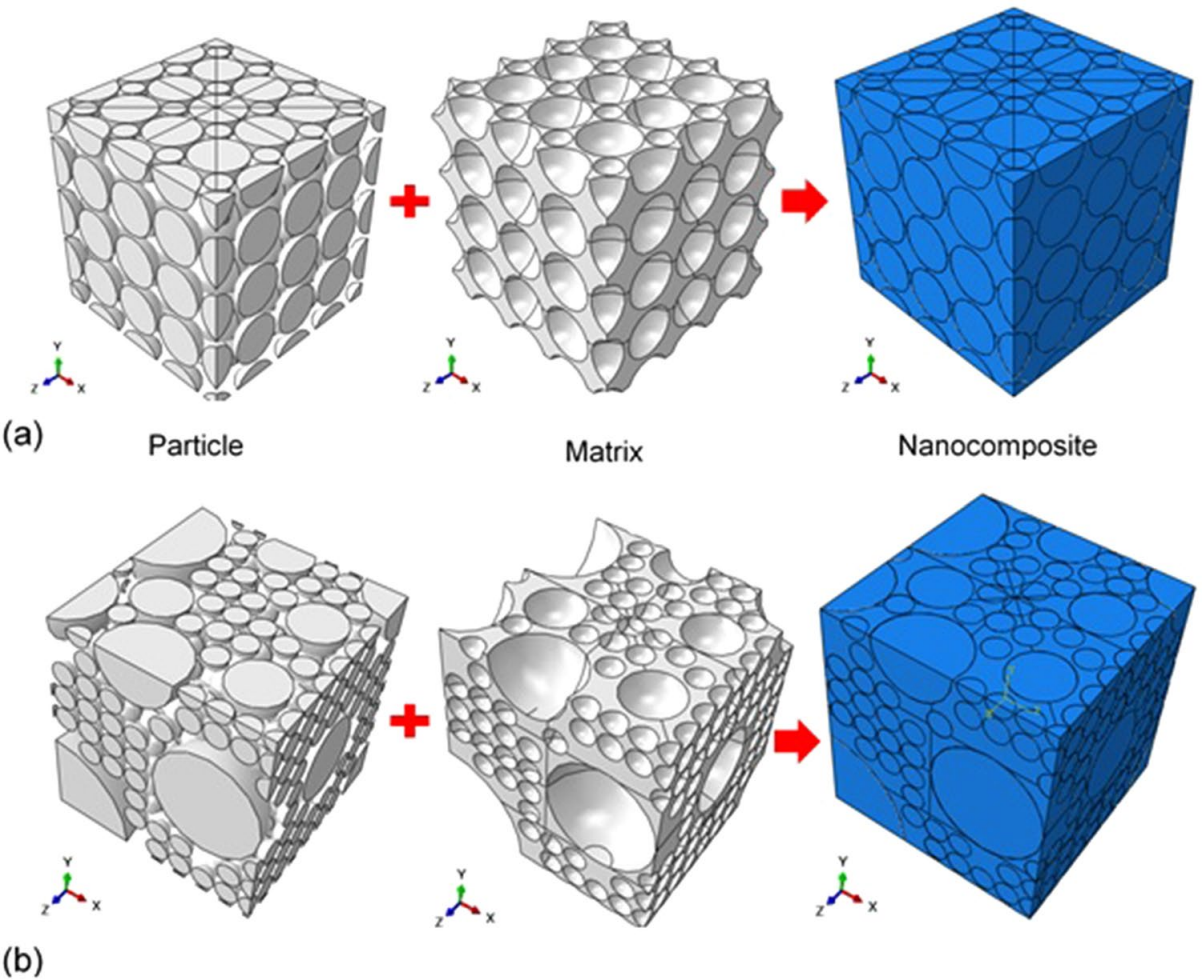
Schematic view of particles, Matrix and nanocomposite in the model of (**a**) regular and (**b**) irregular particle patterns.

A schematic view of the FE models of the nanocomposite is shown in Fig. 4. Regular and irregular particle distributions were considered in the FE modelling such that the particle volume fraction was kept equal to the original condition. Consequently, only the effect of particle distribution was investigated in the nano-indentation experiment of the nanocomposite. In the regular model, many average size particles (i.e. 0.5 μm and 1 μm) were distributed in the form of regular pattern through the cubic block (Fig. 4(a)), while the irregular model was created using different sizes of particles that spread irregularly similar to the particle distribution pattern of the SEM images of the nanocomposite specimen (e.g. Fig. 1) as shown in Fig. 4(b).

**Figure 5:**
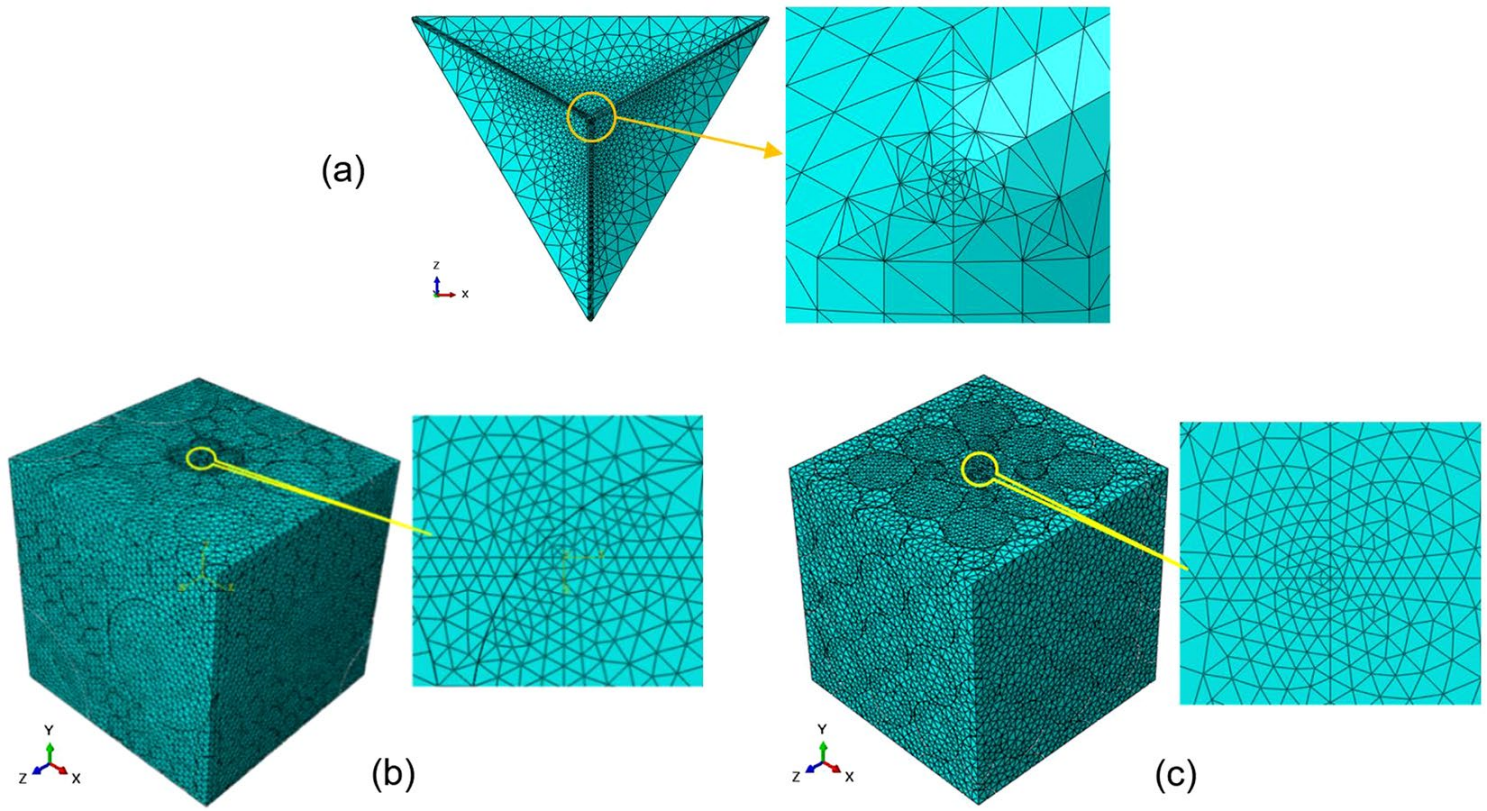
Mesh pattern of (**a**) the indenter, (**b**) the nanocomposite specimen with regular particle distribution and (**c**) with irregular particle distribution

The particles and Matrix parts are created individually, and assembled with a tie constraint between the particle surfaces and the Matrix^39,40^. Four-node linear tetrahedron solid elements (C3D4) were employed to mesh the particles and Matrix while the indenter was meshed using three-node rigid triangular facet elements (R3D3). The elements were refined toward the contact areas of indentation in the specimen and the indenter as shown in Fig. 5. Moreover, a similar size of elements was applied to the indenter tip and the contact area of the specimen, to keep nodes of both parts close to each other and reduce the convergence problems. The element size were considered small enough to minimize the computational error, in which the mesh convergence study has been performed completely^53^.

The indenter was loaded in a displacement control mode similar to the loading condition in the experiment. Therefore, the indenter was moved vertically into the specimen up to the maximum depth of 300 nm in 30 sec, held for 10 sec at the maximum depth and unloaded for 30 sec. The response of the system as the load-displacement curve was measured throughout the FE simulation and compared with the tests. The boundary conditions were imposed by fixing the lateral and bottom surfaces of the specimen. The interaction between the indenter and the specimen was modelled as surface to surface contact with finite sliding in frictionless condition.

**Figure 6:**
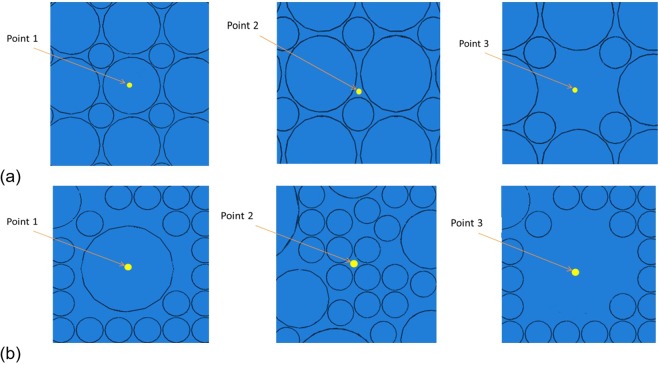
Indentation on different locations on a) regular and b) irregular FE models of the nanocomposite.

The indentations were performed on three different regions of the regular and irregular models with various distributions of particles in order to investigate the effect of indentation on different locations in the results of nano-indentation experiment. The indentation points were selected on a large particle, on the Matrix between the particles and on the Matrix away from the particles in both regular and irregular models as shown in Fig. 6.

### Mechanical properties of different parts of the FE models

Since the indenter applies normal pressure to the sample surface, the resin and particles of the nanocomposite are subjected to compressive stresses. Therefore, compressive elastic-plastic properties were defined for each constituent of the nanocomposite using bi-linear stress-strain curve^54,55^. The mechanical properties of Zirconia ceramic particles and Bis-GMA biopolymer resin were extracted from the literatures^53,56–58^ as listed in Table 1.

**Table 1:** Mechanical properties of different components of the nanocomposite.

	E (GPa)	ν	σ_Y_ (MPa)
Zirconia particles	100	0.32	1200
Bis-GMA resin	1.5	0.4	25
Matrix	4.96	0.37	100.43

The original particle volume fraction of the nanocomposites is 63.3% as reported in the manufacturing data sheet^59^. The SEM images are used to account for the volume fraction of the fine particles (d <0.5 μm) that was not considered in the FE model, as well as the remaining particle and resin volume fractions in the dental nanocomposite as listed in Table 2. The rule of mixtures was used to calculate the mechanical properties of the Matrix region. In this respect, Mori-Tanaka^60^ and Szazdi-Pukanszky^61^ equations were selected based on the particle shape and the volume fraction to determine the elastic modulus and strength of the Matrix as listed in Table 1.

**Table 2:** Volume fractions of resin and particles with diameters less than 0.5 µm in the resin of the nanocomposite.

	V_f_-Nanocomposite	V_f_-Matrix
Resin	36.7%	65%
Fine particles	12.8%	35%
Large particles	50.5%	0%

## Results and Discussion

The experiment and the FE simulation results are described in terms of the global response of the nanocomposite as load-displacement curves and mechanical properties, and also the internal behaviour of the nanocomposites in the form of deformation and plastic strain.

### Experimental

**Figure 7:**
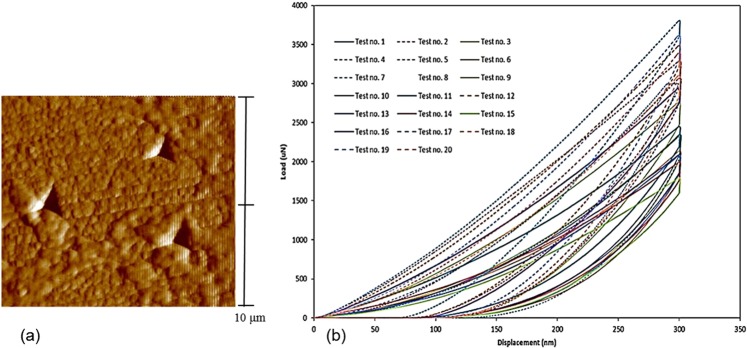
(**a**) A sample of SPM image of the nano-indentation residual impressions on the specimen and (**b**) load-displacement response of nano-indentation test on the dental restorative nanocomposite.

The results of nano-indentation experiments in which a perfect residual impression without any damage or crack was created on the surface of the specimen, were applied to calculate the mechanical properties. In general, the existence of any damage or crack around the indentation impression may cause the values of hardness and elastic modulus obtained from the nano-indentation test, do not represent the mechanical properties of the sample material. Therefore, all SPM images of the nano-indentation residual impressions were controlled in order to confirm the accuracy of the mechanical properties calculated from the experiment. A sample of SPM image in 2D views for three residual impressions on the specimen is shown in Fig. 7(a). According to Bolshakov and Pharr^62^, in the condition that the ratio of final depth to the maximum depth (h_f_/h_max_) is less than 0.7, the effect of pill-up or sink-in is negligible. In this study, the ratios of h_f_/h_max_ obtained from the nano-indentation experiments were less than 0.7, indicating that the pile-up or sink-in does not affect the results. The load-displacement response of the experiment in many randomly selected points is also demonstrated in Fig. 7(b).

**Figure 8:**
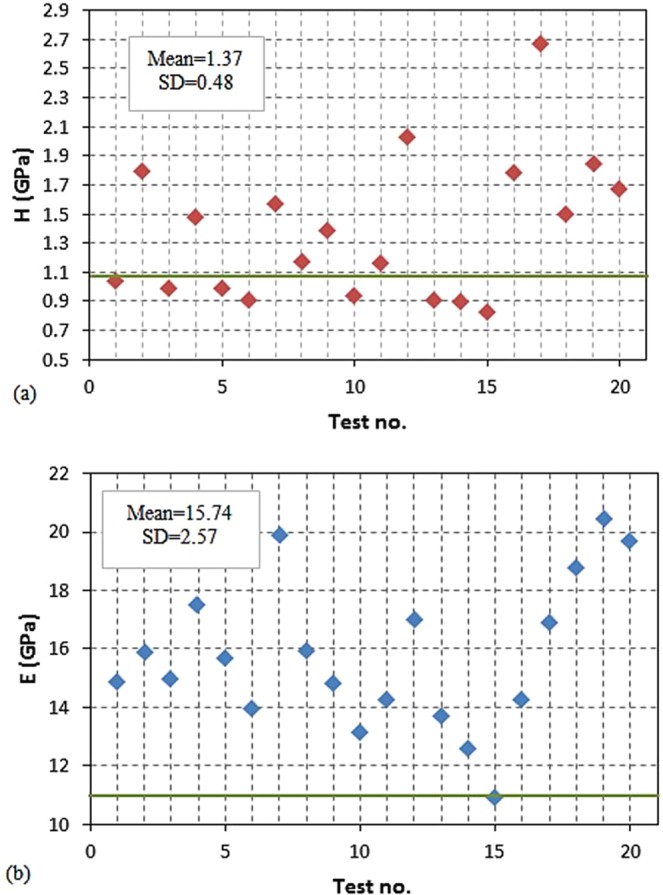
(**a**) Hardness and (**b**) elastic modulus obtained from the nano-indentation experiment on different regions of the dental nanocomposite, the horizontal green lines show the properties of the composite obtained from micro- and macro-scales experiments.

The hardness and elastic modulus properties of the nanocomposite were calculated using the valid results of the nano-indentation tests (Fig. 7(b)) and Eqs (1) and (3). These values were then plotted in diagram form as presented in Fig. 8. The values of properties obtained from micro- and macro-scales experiments are also shown in Fig. 8 using solid lines ^41,42^.

**Figure 9:**
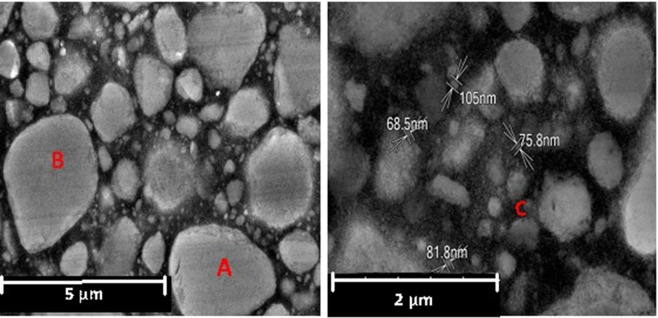
SEM images of the nanocomposite specimen with different magnifications.

The experiment results of the nano-indentation at randomly picked locations in terms of hardness (Fig. 8(a)) and elastic modulus (Fig. 8(b)) properties were obtained in scatter form which is due to the non-homogeneity of the sample material at different indentation points. The material response in the test as load-displacement curve (Fig. 7(b)) is recorded with high variations of maximum load in the range of 1600 μN to 3600 μN. Consequently, the calculated mechanical properties vary from 10.86 GPa to 20.43 GPa for elastic modulus and 0.82 GPa to 2.67 GPa for hardness. Therefore, an assessment is performed on the distribution of particles in the nanocomposite resin, using SEM images with various magnifications that were taken from the specimen surface as shown in Fig. 9. In these figures, the ceramic particles and the polymeric resin are observed in light grey and black colours, respectively.

The SEM images help to understand the high variations in the properties that obtained from the indentation in different locations. In general, indentation on a relatively large particle of the nanocomposite (points A or B, Fig. 9) results in the determination of high values of stiffness and hardness, while the low values obtained from the indentation on the region with fine particles (point C, Fig. 9).

**Figure 10:**
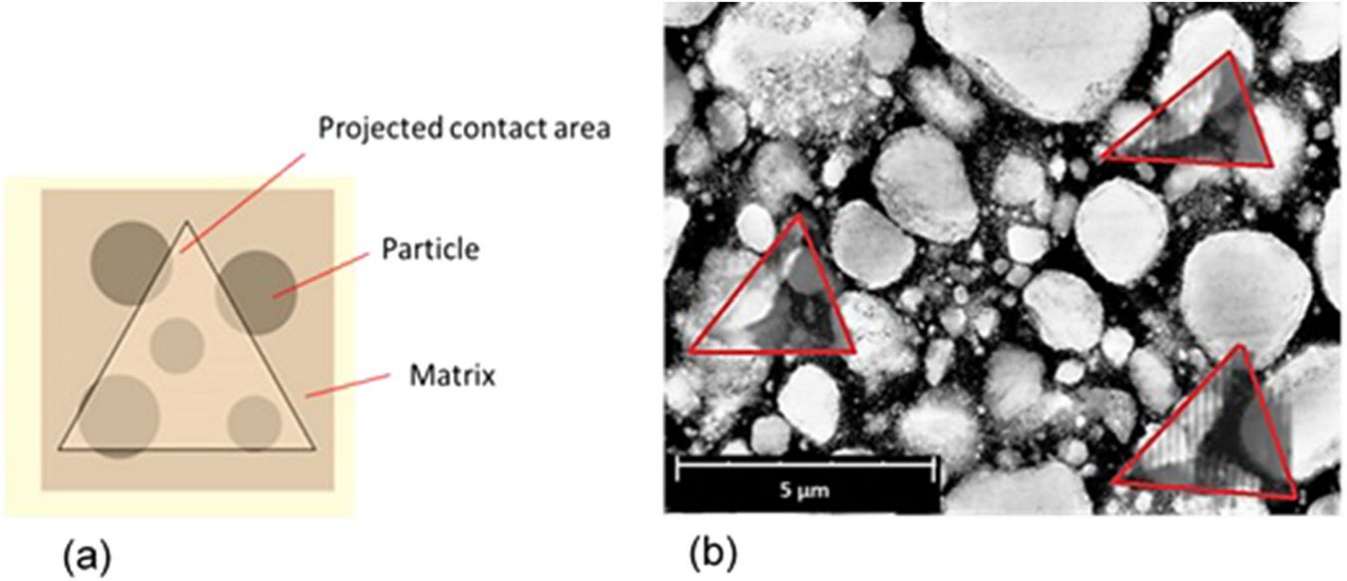
(**a**) Schematic representation of indentation on heterogeneous material, (**b**) overlapping the nano-indentation impression obtained from the SPM image (Fig. 7(a)) with the SEM image of the nanocomposite specimen.

Previous studies about nano-indentation experiment on particle reinforced composites^30–32^ indicated that, if the indenter contacts all components of the non-homogeneous material through the loading as schematically shown in Fig. 10(a), the properties obtained from the nano-indentation experiment could represent the equivalent mechanical properties of the material. Consequently, repeatable load-displacement response is obtained for the indentation on different points. In order to visualize the size of the residual indentation mark of Filtek Z350 XT nanocomposite, the exact size of the 2D SPM image of the nano-indentation impressions (Fig. 7(a)) is measured, duplicated and overlapped on an SEM image of the nanocomposite, as shown in Fig. 10(b).

The residual indentation impressions (red triangles, Fig. 10(b)) cover a considerable volume of resin and particles in different sizes. It should be mentioned that these residual areas were remained after recovering the elastic deformation at the end of unloading stage, and are less than the real contact area between the indenter and the specimen. Therefore, it was expected that the nano-indentation experiment reports the equivalent mechanical properties of the nanocomposite specimen as reported in the previous studies^30–32^. Although the indentation impression could contain a considerable share of particles and resin, the results of the nano-indentation on the nanocomposite in the form of load-displacement curves and mechanical properties were dispersed. Moreover, a significant difference was observed between properties acquired from the nano-indentation test and those obtained from micro- and macro-scales experiments which are shown by a solid line in Fig. 8(a,b)^63,64^. The estimated errors of the mean values of hardness and elastic modulus obtained from the nano-indentation test were 26.8 and 48.5 percent compared to the micro- and macro-scale tests, respectively.

### FE simulation

**Figure 11:**
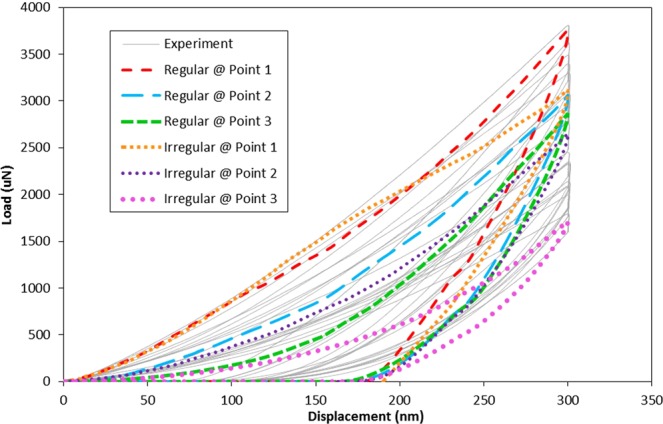
Load-displacement curves obtained from the nano-indentation simulation along with the experimental data.

The FE simulation of the nanocomposite was used to provide insight into the mechanism of deformation and variation of plastic strain through the nano-indentation experiment. In this respect, the FE results in terms of normal load applied to the indenter tip corresponding to its vertical movement were computed for the simulation of the indentation at six different points (Fig. 6) of the regular and irregular models of particle distribution (Fig. 4). Then, the FE results in comparison with the range of load-displacement data that measured from the twenty nano-indentation tests at different locations are shown in Fig. 11. The predicted load-displacement curves of the nanocomposite are in the range of the curves obtained from the experiment, which implies to the validity and accuracy of the FE model and simulation process. The maximum load range was predicted 1713.49 μN to 3761.22 μN, which are in agreement with the corresponding experiment results of 1784.78 μN to 3792.47 μN.

The FE results indicated that the load-displacement data varies with respect to the indentation location on the nanocomposite specimen. In both regular and irregular models, the maximum load and the initial slope of unloading curve of the indentation in point 1 (Fig. 6) are higher than the corresponding values of indentation in points 2 and 3. Such variations indicate the dependency of the nano-indentation result to the location of the indentation with respect to the configuration of the particles and resin in the indenter projected area. These results reveal that the dispersion of the values of hardness and elastic modulus obtained from the experiment is due to the heterogeneous nature of the nanocomposite specimen in the scale of the nano-indentation test. Moreover, the large difference between the mechanical properties of the constituent phases of the nanocomposite (i.e. ceramic particles and polymer resin) enhances the dispersion of the response of the nano-indentation experiment at different regions. It should be noted that, the lack of information about the exact location of indentation through experiment caused discrepancies between the test and the simulation data.

The hardness values of the nanocomposite were computed according to the Karimzadeh, *et al*.^65^, which was obtained from 1.02 GPa to 2.40 GPa for indentations at different locations. These values agree well with the hardness values of 0.87 GPa to 2.67 GPa that was obtained through experiment.

**Figure 12:**
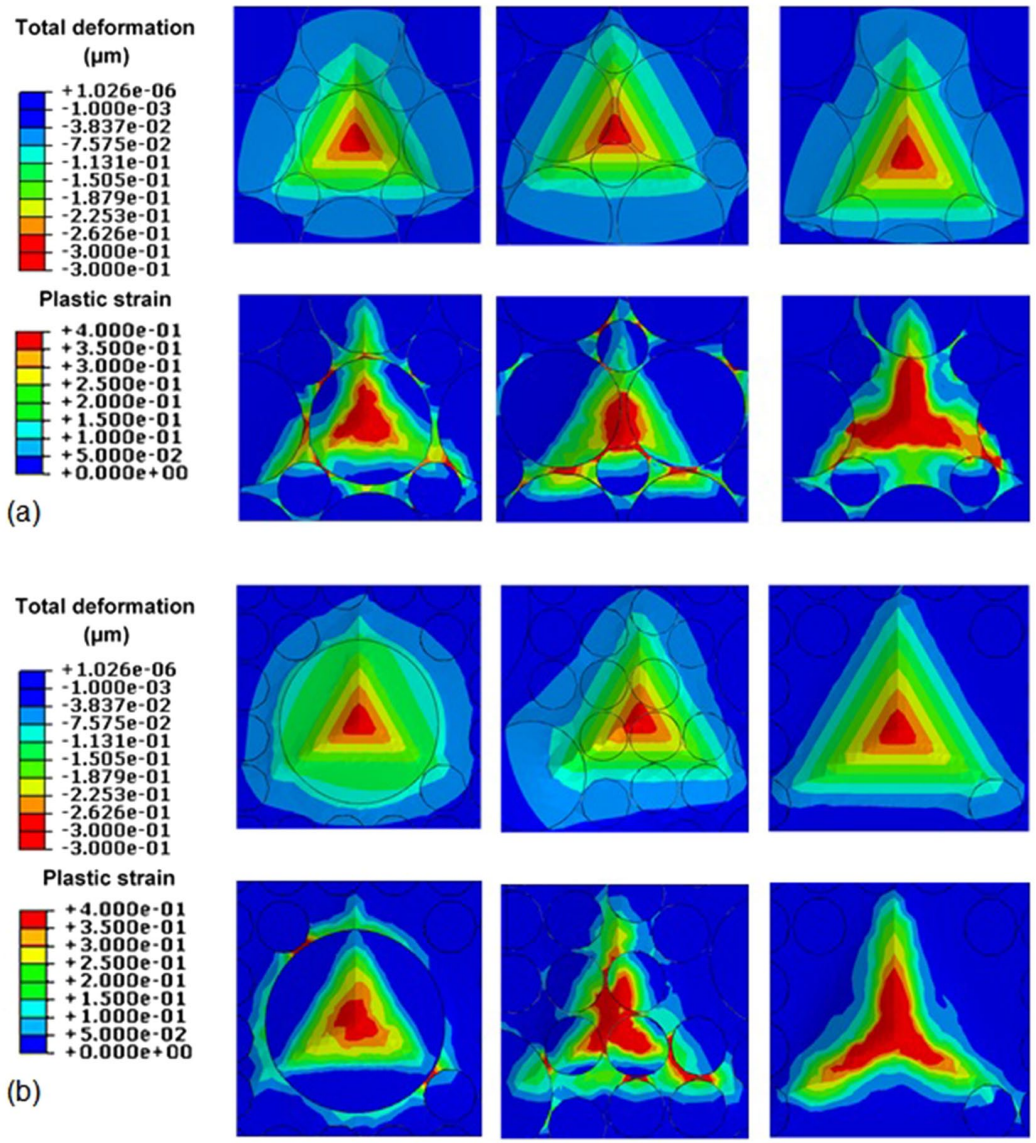
Total deformations and plastic strain of the specimen beneath the indenter at maximum load for indentation on different points of (**a**) regular and (**b**) irregular models of particle distributions.

The total deformation and plastic strain of the specimen beneath the indenter obtained from indentation on different points of regular and irregular models at maximum load (i.e. at the indenter penetration of 300 nm) are demonstrated in Fig. 12. The surface of the specimen, where is in contact with the indenter tip, can be observed as a triangular area in Fig. 12, similar to the indentation impressions represented in Fig. 7(a). Results indicated that the indenter is in contact with a series of particles and Matrix in all models which contributed in the disordered formation of the equilateral indentation mark. The contour plot of the plastic strain which computed in the form of disordered shape reflects the effect of high stiffness/strength particles that are loaded under the Berkovich loading indenter. Such strain formation indicates the event of stress locality phenomena with different magnitudes that randomly varied and depended to the volume and configuration of the particles.

On the other hand, the projected contact area between the indenter and the specimen (i.e. *A* in Eqs (1) and (2)) is calculated based on a symmetric shape of the indentation impression^1^. While, according to the deformation of the nanocomposite sample beneath the indenter (Fig. 12), the contact area between the indenter and the specimen is not symmetric even in the model with the regular distribution of the particles. Besides, precise viewing of the SPM images taken from the indentation impression (Fig. 7(a)) indicates the asymmetric residual contact area which verifies the simulation results. Therefore, in the case of indentation on the heterogeneous materials the projected contact area of the sample and the indenter should be calculated based on the asymmetric shape of the indentation impression in order to provide accurate results.

It is worth to mention that, to measure the mechanical properties of a heterogeneous material by the nano-indentation test, the specimen contact area with the indenter should be a representative volume element of the material. However, defining the representative volume element of the specimen is not applicable for some non-homogeneous materials consisted of high volume fraction of particles of various diameters distributed throughout a resin without a specific pattern, such as the dental nanocomposite used in this study.

## Conclusion and Remarks

A large number of nano-indentation experiments were performed in randomly selected regions of Filtek Z350 XT, nano-particle reinforced dental nanocomposite, as a heterogeneous material. The results of the nano-indentation experiment in terms of hardness and elastic modulus properties displayed very dispersed values, in which the measured average properties showed 26.8 and 48.5 percent errors, respectively, compared to the nanocomposite properties obtained from the macro-scale tests. In order to interpret the results of the experiment, a hybrid computational-experimental approach was developed. Therefore, several representative FE models of the nanocomposite micro-scale configuration were created, in which the results were used to link the internal mechanical behaviour of the material to its global response during the nano-indentation test. The simulation results of the nano-indentation experiment on the dental nanocomposite verified the experimental observation. The hardness values were predicted from 1.02 GPa to 2.40 GPa, which are in the range the experimental values obtained from 0.87 GPa to 2.67 GPa. The contour plot of the deformation and the plastic strain highlighted the stress locality phenomenon that caused by high strength particles which distributed randomly in different configurations beneath the Berkovich indenter tip. The simulation results revealed that the contact area of the specimen with the indenter was an asymmetric triangle instead of an equilateral triangle. Therefore, it was suggested to modify the nano-indentation formulation based on the asymmetric projected contact area to acquire precise properties of heterogeneous materials using the nano-indentation experiment. The hybrid approach is applicable for evaluation of the nano-indentation method in characterization of other nanocomposite materials with different particle sizes.

## Data Availability

The datasets presented in this paper are available from the corresponding author A. Karimzadeh on reasonable request.
